# Functional group diversity increases with modularity in complex food webs

**DOI:** 10.1038/ncomms8379

**Published:** 2015-06-10

**Authors:** D. Montoya, M.L. Yallop, J. Memmott

**Affiliations:** 1 School of Biological Sciences, Life Sciences Building, University of Bristol, 24 Tyndall Avenue, Bristol BS8 1TQ, UK

## Abstract

Biodiversity increases the ability of ecosystems to provide multiple functions. Most studies report a positive relationship between species richness and the number of ecosystem functions. However, it is not known whether the number of functional groups is related to the structure of the underlying species interaction network. Here we present food web data from 115 salt marsh islands and show that network structure is associated with the number of functional groups present. Functional group diversity is heterogeneously distributed across spatial scales, with some islands hosting more functional groups than others. Functional groups form modules within the community so that food webs with more modular architectures have more functional group diversity. Further, in communities with different interaction types, modularity can be seen as the multifunctional equivalent of trophic complementarity. Collectively, these findings reveal spatial heterogeneity in the number of functional groups that emerges from patterns in the structure of the food web.

## Abstract (web summary)

The influence of functional group diversity on food web structure is less well known than that of biodiversity. Analysing species interactions in a network of salt marsh islands, Montoya *et al*. show that functional group diversity is higher in more modular networks and varies spatially across the archipelago.

## Graphical abstract

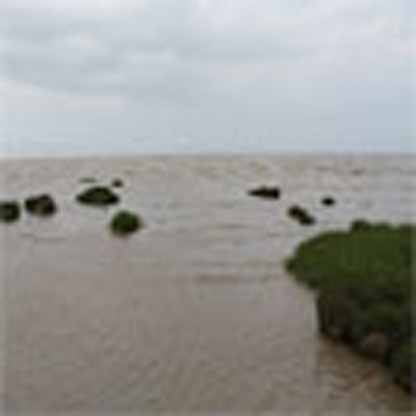

The last two decades of ecological research have revealed consistent relationships between structure and function in ecological communities, and have demonstrated that species richness is important to understanding how ecosystems function^1^,^2^,^3^,^4^,^5^,^6^,^7^. In recent times, there has been a growing interest in functional traits and functional groups versus species, mainly because functional group diversity is more meaningful than species diversity and it is associated with greater long-term stability^8^,^9^. Food-web approaches consider both species diversity and the fluxes of energy and materials between species through their interactions, thus providing a natural framework for understanding species' ecological roles and the mechanisms through which biodiversity influences the number and the distribution of functional groups in ecological communities. Despite this, the vast majority of studies consider parameters such as species richness and study a single trophic level^2^,^5^,^6^,^10^,^11^,^12^. However, it is increasingly clear that we need to consider the interactions between species and include more than one trophic level in our studies, because these will have a profound effect on our ability to conserve, restore or manage ecosystems^13^. This involves looking at how species interactions are organized (network structure), how they contribute to functional group diversity and how species from different functional groups affect each other, as these will interact indirectly via shared host plants, prey species or natural enemies^14^,^15^.

Another dimension often overlooked in studies linking community structure and functional group diversity is space, that is, how does spatial variation in community structure affect the number of functional groups? Ecological communities are assembled according to processes that operate over a large range of spatial and temporal scales, including the historical sequence of colonization and extinction events, environmental filtering, local species interactions and phylogeny^16^,^17^,^18^,^19^,^20^. These assembly processes determine a community's extant structure^21^,^22^, that is, the species present in a local community, which in turn shape the structure of species interactions. At larger, metacommunity spatial scales, local communities can differ in their species composition and, as shown by empirical studies incorporating spatial variability, this spatial segregation of taxa can influence food web topology^23^. The central challenge thus consists of determining which aspects of diversity and food-web structure are related to functional group diversity. Whereas the literature on the effects of species richness is vast, little is known about how changes in the structure of species interactions affect functional group diversity. As many network properties systematically vary with the size and complexity of biological communities, we expect spatial changes in food web structure to be common, and that these will be likely to result in spatial heterogeneity in the number and distribution of functional groups. Understanding these patterns is critical in determining relationships between network structure and functional group diversity in complex communities.

Aside from a few non-experimental studies^24^,^25^, the vast majority of data on the relationship between community structure and function come from controlled, small-scale experiments using field plots or laboratory experiments^12^,^26^,^27^,^28^. The extrapolation of results from rigorous experimental studies with low spatial heterogeneity to natural and more complex ecosystems with high spatial heterogeneity is not straightforward though. To manage and conserve ecosystems, it is essential to understand the spatial variability of the biological communities hosting the diversity of functional groups. Here we use a model field system to ask whether the structure of species interaction networks varies spatially and whether this is associated with spatial heterogeneity in the number of consumer-resource functional groups, that is, functional group diversity. If it does, this has important implications for the conservation of multifunctional ecosystems in real-world communities.

Our model system consisted of 115 small salt marsh islands located in four archipelagos, each comprising 27–31 islands, 0.2–52.4 m^2^ in size, on intertidal mudflats (Fig. 1). We constructed a food web for each island and used these to examine the relationship between food-web structure and six functional groups defined by different types of interaction within the food web. We sampled all eukaryotic organisms on each island with the exception of nematodes: these comprising terrestrial plants, marine macrophytes, diatoms, crustaceans, molluscs, spiders and insects, the latter including pollinators, decomposers and a predator. Interactions between species were determined by a mixture of direct observation, gut content analysis, stable isotope analysis, literature searches and discussion with experts. We characterized qualitatively the ecosystem functions provided by the species inhabiting the archipelagos (namely, primary productivity, habitat provision, herbivory, pollination, predation and decomposition) and looked at the number of consumer-resource functional groups providing those functions that were present on each island. We then examined the distribution of the number of functional groups within (local scale) and between (regional scale) archipelagos. Species in natural communities can contribute to one or more functional groups (for example, terrestrial plants are involved in primary productivity and habitat provision); if several species belong to a single functional group, then those species are redundant (for example, diatoms, marine macrophytes and terrestrial plants are involved in primary productivity; several insects contribute to pollination). It is important to note that in this study system only a few species contributed to more than one functional group and each functional group comprises at least two species. As species richness and food-web properties are not completely correlated^29^, this allows testing their relative importance in determining the number of functional groups.

Our analysis indicates that complex food webs of different interaction types show spatial variation in network structure, a pattern that is observed both within and across archipelagos of salt marsh islands. This spatial variation in network structure is associated with the number and distribution of functional groups within the overall community. In particular, more modular architectures are associated with higher functional group diversity. These results show that in communities with multiple types of interactions or ‘networks of networks', modularity can be seen as the multifunctional equivalent of trophic complementarity (TC). In the Anthropocene where most natural systems are fragmented and patchily distributed in space, these results have important implications for the conservation and management of multifunctional ecosystems.

## Results

### Spatial patterns in network and functional group diversity

**Figure 2:**
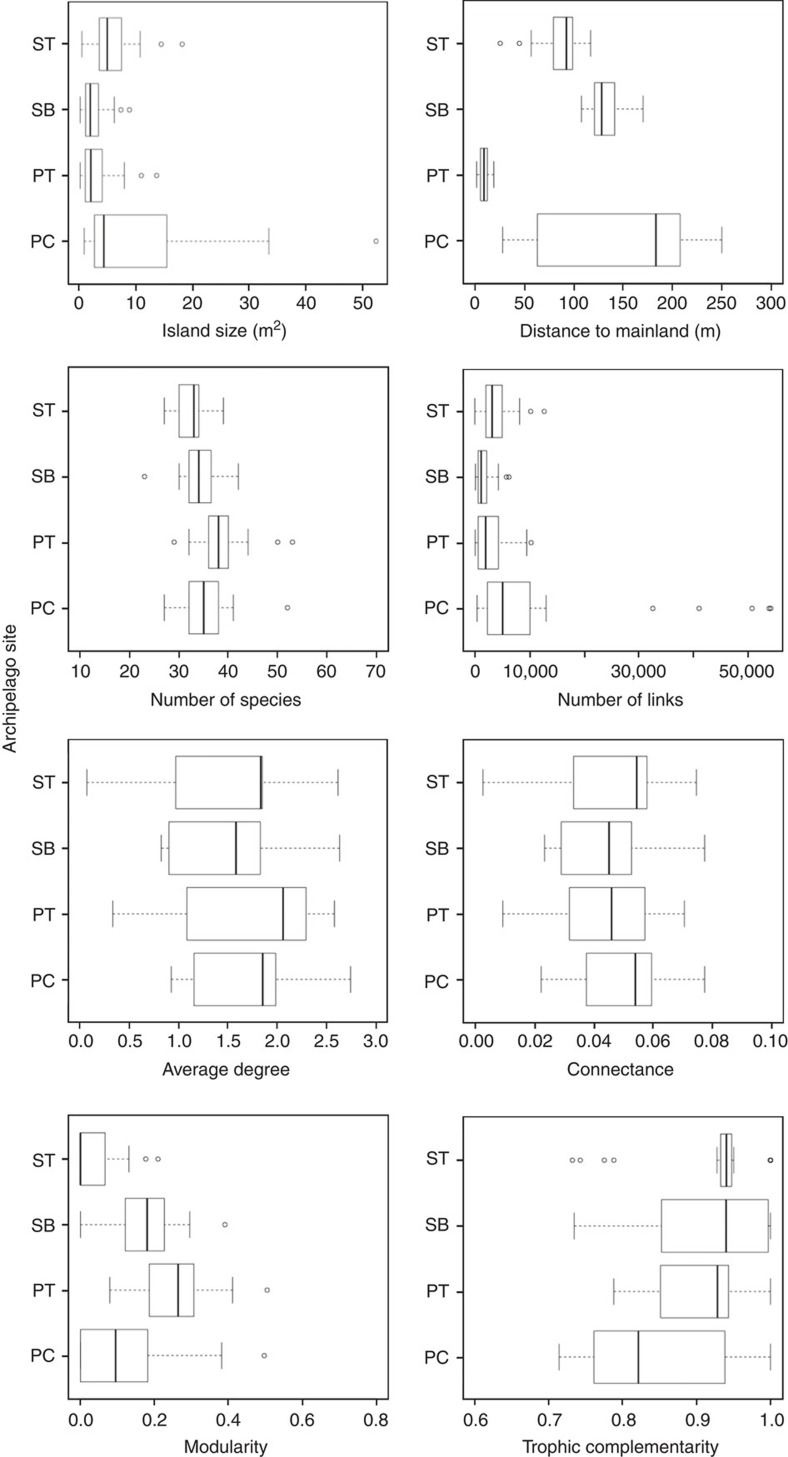
Network and biogeographical properties. Boxplots showing the range of values for network and biogeographical variables. Network metrics include the following: species richness (*S*), number of links (*L*), average degree (*L/S*), connectance (*L/S*^*2*^), modularity (degree to which interactions occur more frequently within modules than between modules) and trophic complementarity (based on nestedness, which measures the degree to which the diets of consumers are proper subsets of other, more generalist consumers). Results are given for the four archipelago sites. ST=Steart (*N*=29); SB=Sand Bay (*N*=31); PT=Portishead (*N*=27); PC=Poet's Corner (*N*=28). The boxes represent the median (black middle line) limited by the 25th (Q1) and 75th (Q3) percentiles. The whiskers are the upper and lower adjacent values, which are the most extreme values within Q3+1.5(Q3−Q1) and Q1−1.5(Q3−Q1), respectively. Points outside this interval are represented as dots.

The network properties of the salt marsh communities fall within the range of values reported in food-web studies considering multiple interaction types^15^. These communities showed a high spatial heterogeneity in their structure, in terms of species numbers, number of links, linkage density, connectivity of the network and modularity patterns (Fig. 2). Spatial differences in network structure were detected both within and across archipelagos. Most empirical food webs do not include spatial variation in their structure and these results add to the few studies documenting spatial heterogeneity in food-web patterns^23^.

**Figure 3:**
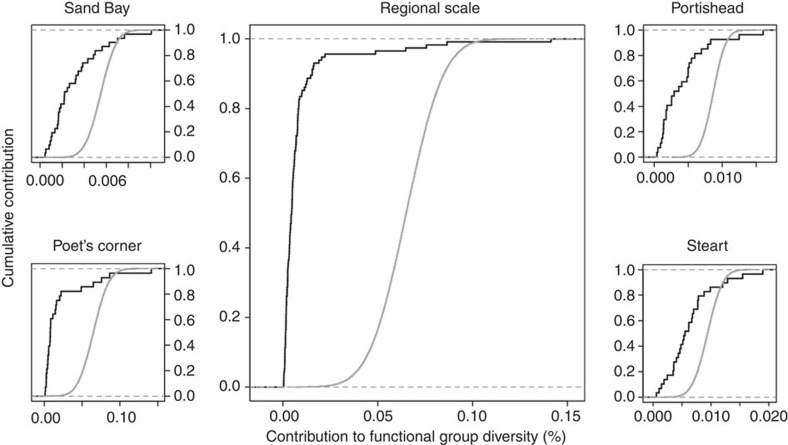
Empirical cumulative distributions of the contribution of each island to functional group diversity. The contribution to functional group diversity, that is, number of functional groups, is calculated using species abundance data, assuming all individuals within species contribute equally. Data are provided for each archipelago individually: the black line represents the field data and the grey line represents the empirical cumulative distribution of a normal distribution with the same mean and s.d. If all islands contributed equally, a straight vertical line would be observed in these plots with a value *x*=1/number of islands (Sand Bay=0.032, Portishead=0.037, Poet's Corner=0.036, Steart=0.034). The contribution to functional group diversity is heterogeneously distributed among islands within and across archipelagos, with most islands contributing less than expected and only a few islands contributing fully to functional group diversity.

In contrast to a null expectation where all islands contribute equally to functional group diversity (or where the diversity of functional groups is normally distributed), the number of functional groups was heterogeneously distributed in space. The average number of functional groups per island was 4.35 (s.d.=±0.88) and only 12.17% of the islands hosted all functional groups. This pattern was consistent within and between archipelagos, and was seen for both species abundance (Fig. 3) and species richness data (Supplementary Fig. 2). Thus, some islands in our saltmarshes have higher functional group diversity than others. These results add to and complement the concept of functionally important species reported in previous studies (that is, the observation that not all species are functionally equivalent)^5^, by adding a spatial component (that is, not all islands contribute equally to functional group diversity).

### Drivers of spatial variability in functional group diversity

Next, we explored the relationship between the structure of island food webs and the number of functional groups provided by each island. We used spatial generalized estimating equations^30^ to identify the most important set of properties—species richness, abundance, number of links, connectance, modularity, linkage density, island size and distance to mainland—that were related to functional group diversity. Functional group diversity was favoured in islands whose food webs presented more modular architectures; this pattern being robust irrespective of the archipelago or spatial scale considered (Table 1). Different interaction types formed modules in our salt marsh communities, this underpinning the division of ‘networks of networks' into their separate component networks (for example, pollination networks and herbivory networks) for the quantitative study of individual functions. Depending on the archipelago considered, the models show that linkage density, connectance, number of links and distance to mainland are also correlated with the number of functional groups. At the regional scale, the distance to mainland was negatively correlated to the number of functional groups (Wald test, *N*=115, *P*=0.005), suggesting that proximity to mainland benefits functional group diversity. For example, pollination, which is provided by up to 38 insect species in our system and is exclusive to terrestrial ecosystems, is found only on islands close to land. It is unclear although, whether this is a consequence of limits in insect flight range or the ability of terrestrial plants to tolerate seawater. Increasing distance to mainland favoured functional group diversity in one of the archipelagos though; hence, the association between proximity to mainland and functional group diversity is not clear.

### Topological role of species and functional group diversity

**Figure 4:**
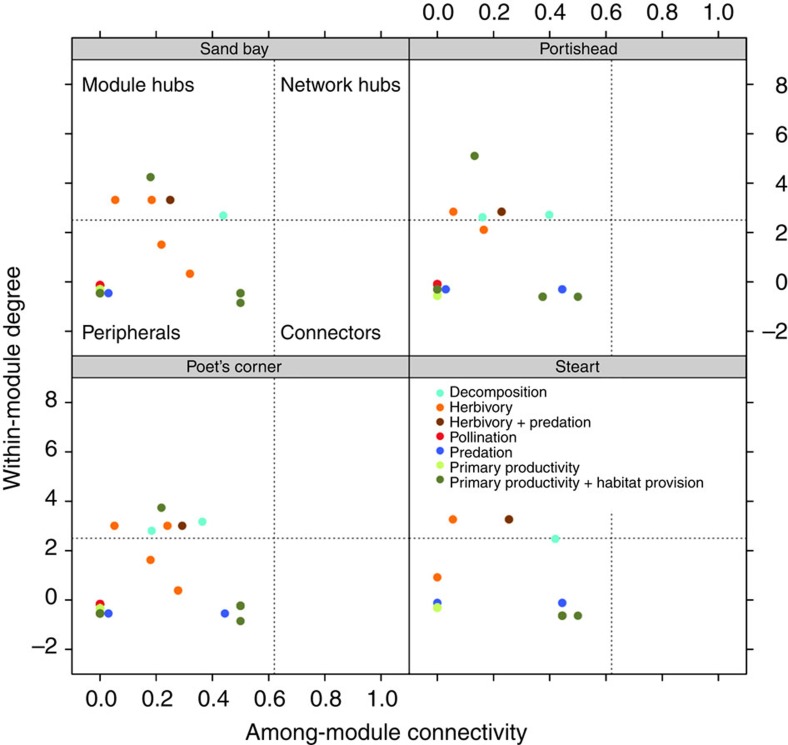
Distribution of functional group diversity according to the species' network role. Circles represent functional groups of species in the food web; species functional group is differentiated by colour. The four archipelagos are shown and the dotted lines represent the threshold values for classification of species/functional groups into the four categories^32^.

To further understand the distribution of functional groups within the food webs, we assigned a role to each species according to its topological network properties^31^,^32^. This method helps identify potential relationships between functional groups and species topology, thus improving our understanding of how the locations of individual species within a network relate to their functional role. Species had similar network roles within and across archipelagos (Fig. 4), and they fell into two categories: peripheral species (diatoms, insect pollinators, most terrestrial plants, *Littorina* sp., *Linyphiidae* sp., *Symplecta stictica* and *Cillenus lateralis*), and module hubs (*Anurida maritima*, *Carcinus maenas*, *Gammarus salinus*, *Lekanosphaera rugicauda*, *Aster tripolium* and *Hydrobia ulvae*). Primary productivity, habitat provision, herbivory and predation comprised species in both categories. Pollination comprised only peripheral species (although only one flowering plant was present on the islands), whereas module hubs were responsible for decomposition (the Collembola *A. maritima* had the highest among-module connectivity). Several studies have reported that community modules are connected to each other by relatively few ‘hub' species and it is these species that are most structurally important for the robustness of the community overall (for example, one study reported that only 15% of the species in pollination networks were structurally important^32^). Further, the re-establishment of structurally important species that link different community modules is a fundamental step towards restoring robust, resilient communities at the landscape, metacommunity scale^33^. This suggests a stabilizing role of decomposer species *A. maritima* acting as a community hub in our island salt marsh systems.

If functional groups are arranged into modules, we would expect individual species of similar functional groups in the network to show a very low number of interactions with species in other modules (that is, low among-module connectivity). The topological analysis detected reduced connectivity values among species of different community modules, this indicating low overlapping between functional groups. The absence of species with high among-module connectivity further supports our finding that in natural communities, functional groups are arranged into modules.

### Trophic island biogeography

In addition to the effects of network architecture, the number and distribution of functional groups are likely to be affected by gradients in biogeographic variables and the interactions between these need to be understood before any assessment of the response of functional group diversity can be undertaken. This is a central tenet of the Trophic Island Biogeography theory, which argues that area (that is, island size) and distance to mainland control food-web properties such as connectance and the number of links^34^. Although there were some statistically significant correlations (Supplementary Tables 3 and 4), the reported significant relationships between biogeographic variables and food-web properties were not consistent among archipelagos or affected by species origin (terrestrial or marine). Thus, our results do not universally support Trophic Island Biogeography predictions, and show that at the scale of our study system, species co-occurrences and food-web properties vary independently of biogeographic variables such as island size and distance to mainland.

### TC analysis

**Figure 5:**
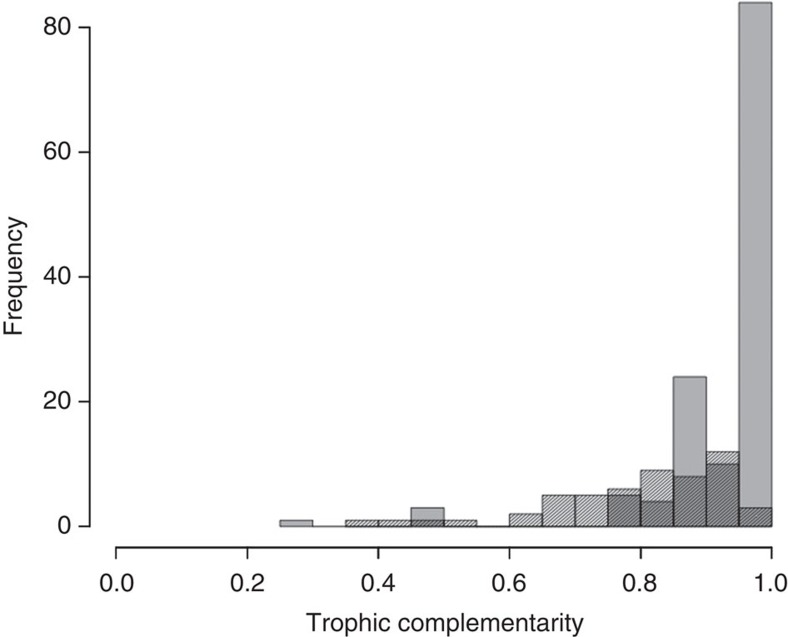
Frequency distribution of trophic complementarity values across functional groups. Trophic complementarity is calculated following Poisot *et al*.^35^. Grey bars correspond to bipartite consumer-resource functional groups in our salt marsh system (plant-herbivory webs, decomposer webs and prey-predator webs). Bars with shaded lines correspond to trophic complementarity values of 54 plant–pollinator networks from the literature^55^.

We divided the salt marsh communities into their functional components or modules and estimated TC within them. TC is based on niche partitioning and it has been shown to be an ‘important mechanism driving functioning in multi-trophic communities'^35^,^36^. We calculated TC for all consumer-resource functional modules in the salt marsh system. TC varied across functional groups, with a general tendency towards high values, a result further supported with data from other plant-pollination networks from the literature (Fig. 5). Collectively, these results contribute significantly to our understanding on the relationships between functional group diversity and food-web structure. Thus, scaling up to the global salt marsh community (the four archipelagos together), modularity can be seen as the multifunctional equivalent of TC, as different functional groups use different resources and form separate sub-networks within the overall community.

## Discussion

The results reported here indicate that to achieve a greater understanding of the relationship between biodiversity, community structure and functional group diversity, the next generation of studies will benefit from adopting a food-web approach^11^. Indeed, the positive biodiversity–function relationship within trophic levels, consistently reported in studies measuring functions quantitatively, is underpinned by niche partitioning, a concept that is implicit in the niche model of food-web structure^37^. Our results do not contradict previous studies demonstrating the importance of species richness to the functioning of ecosystems, rather they show that species richness and food-web modularity are non-exclusive drivers of the observed spatial patterns in functional group diversity. There was no consistent relationship between species richness and modularity in our archipelagos and, for those archipelagos where the relationship was statistically significant, the variability in modularity explained by species richness was low (*R*^2^_Regional_=0.15, *P*<0.01; *R*^2^_Poet's Corner_=0.12, *P*=0.04; see also Supplementary Table 4). This shows that although the provision of multiple functions requires greater numbers of species, as demonstrated by previous quantitative experiments^2^,^4^,^5^,^6^,^7^,^10^,^11^,^12^, the particular arrangement of species into modules is also determinant to understand the diversity of functional groups, as modules separate functional groups and contribute to functional group diversity in biological communities^31^,^32^,^38^,^39^. Both community characteristics—species richness and food-web modularity—have been associated with the stability of biological communities through effects on redundancy and resistance to perturbations, respectively^40^,^41^,^42^.

Whatever the mechanisms involved, this study reveals spatial heterogeneity in the number and distribution of functional groups (that is, functional group diversity) that emerges from the underlying food-web structure. We were able to identify food-web architectures, specifically modularity, which favour functional group diversity. Functional groups form network modules, this justifying the widespread division of natural communities into their functional components or sub-networks to further study individual functions. However, the current conservation paradigm aims at managing and conserving multifunctional ecosystems, and this demands consideration of ‘network of networks' approaches and the study of how modules and functional groups assemble in the overall community. These results point to the maintenance and restoration of community modules as a cornerstone of conservation policy. They also suggest that if it is not possible to conserve all islands, then conservation efforts should target those islands that are most important for maintaining multiple functional groups. Although the island salt marshes are a model system, our expectation is that any system that comprises a mosaic of habitat fragments, islands or patches will show similar results. Habitat fragmentation is widespread in the Anthropocene and understanding its impact on both ecosystem structure and ecosystem function is an important step in ameliorating its impact.

## Methods

### Details of the study site

The four study sites are archipelagos of salt marsh islands located in the intertidal mudflats along the Bristol Channel in the southwest of England. Each archipelago contained 27–31 islands/patches (with a total of 115 islands), making this study site a naturally replicated system with replication both within and among archipelagos. The four sites were selected to share similar physical and environmental characteristics^1^,^2^,^3^,^4^,^5^,^6^,^7^.

Island size ranged from 0.2 to 52.4 m^2^, this comprising two orders of magnitude, and islands were located 1.33–249.9 m from a mainland salt marsh. The colonization of these islands takes place in two stages: first, photosynthetic organisms, such as marine diatoms and green algae grow on the surface of sediments. This creates mounds that are resistant to erosion at high tides, which are colonized by other marine macrophytes and terrestrial plants, which together with the diatoms and algae, provide resources for invertebrate species.

### Island sieving

Before systematic sampling, a whole island was destructively sampled and sieved using a 1-mm mesh. This provided a species list of the range of organisms that commonly inhabit the salt marsh islands. It also supported our sampling and estimation protocols, as our estimated number of species and abundances were very similar to the numbers found during our destructive sampling. Given their location in intertidal mudflats, salt marsh islands host both terrestrial and marine species, and obtaining estimates of species richness and abundance involved a range of different sampling approaches. The construction of the species interaction networks is described below, with the fieldwork taking place April–October 2013. For each group, abundance was scaled up to provide a total per island, summed across-islands (to give a total per archipelago) and summed across archipelagos to give a regional island salt marsh network. We sampled each island twice during the field season.

### Quantifying marine macrophytes and terrestrial plants

The percentage cover of marine macrophytes (green, yellow–green and brown algae) and terrestrial plants were quantified for each island. For the smaller islands, the percentage cover was straightforward to estimate directly; for larger islands, we used randomly placed quadrats. The number of quadrats was proportional to island size. On each island, marine macrophytes and terrestrial plants present were identified to species and given an abundance measure of 1–4 (following Gibson *et al*.)^43^. Category 1 species were rare, only present once to a few times in the whole island (≤5%). Category 2 species were present in high-enough numbers to be seen easily but occupied ≤10% of the island. Category 3 species could be seen throughout the whole island but occupied ≤50% of the island. Category 4 were the most abundant species occupying >50% of the island.

### Quantifying diatoms

Epipelic free-living diatoms, together with some non-motile species associated with sand grains and seawater, were sampled in the field by placing lens tissue on the mud surface. This approach takes advantage of the vertical migratory behaviour of epipelic diatoms in the sediment^44^,^45^ and the diatoms migrate into the tissue^46^,^47^. The number of samples was proportional to island size and to the heterogeneity of island topography. Samples were stored in Lugol's iodine in the dark to avoid evaporation or volatilization of the chemical. Diatom samples were cleaned using concentrated hydrochloric acid and potassium permanganate digest to remove organic matter to enable identification of cleaned frustules (valves). After cleaning, samples were mounted on microscope slides using Naphrax and were then ready for identification. The samples were pooled for each island and the relative abundance of diatoms on each island was estimated by counting 300 valves using light microscopy (Leitz Orthoplan, original magnification × 1,000), this being the standard approach when sampling diatoms^44^,^45^. All diatoms were identified to species, with reference to standard keys, when possible, otherwise they were morphotyped and each species given an abundance measure of 1–4. Category 1 diatoms were rare, estimated as forming ≤1% relative abundance on the island. Category 2 diatoms had an estimated relative abundance between 1 and 30% on the island. Category 3 diatoms were abundant in the sample with an estimated relative abundance of between 30 and 50% on the island. Category 4 diatoms were the most abundant occupying >50% of the sample on the island.

### Quantifying marine invertebrates

The marine invertebrates found on the salt marsh islands consisted of three species of gastropod snails (*H. ulvae*, *Littorina littorea* and *Littorina obtusata*) and three crustaceans (an amphipod (*G. salinus*), a decapod (*C. maenas*) and an isopod (*L. rugicauda*)). We used quadrats and searched for these species within them. The number of quadrats was proportional to island size and to the heterogeneity of island topography. Given the behaviour of some species (for example, hiding underneath vegetation to avoid desiccation at low tide), vegetation within quadrats was carefully moved when searching for invertebrates. Counts of each species of marine invertebrates were scaled up to provide an estimate of total abundance per island.

### Quantifying insect pollinators

Islands that had flowering plants (30% of islands) were sampled for flower visitors using timed observations. Each timed observation was for 30 min. The number of timed observations depended on the spatial clustering of flowering plants. Usually, most flowers could be observed at once; for larger islands, this was not always possible and we conducted more than one timed observation (each 30 min long) to capture the natural variation within them. Flower visiting insects were collected, identified by a taxonomist, and the plant species visited was identified.

### Quantifying terrestrial invertebrates other than pollinators

The terrestrial non-pollinating invertebrates inhabiting salt marsh islands consist of a species of Collembola (*A. maritima*), a carabid beetle (*C. lateralis*), money spiders (*Linyphiidae* spp.) and a crane fly (*S. stictica*). To quantify Collembola, beetles and spiders quadrats were used and counts scaled up to provide an estimate of total abundance per island. Adults of the crane fly were not seen, rather this species was sampled as a soil-dwelling larvae. To estimate its abundance, we took three randomly placed soil cores (10 cm × 2.5 cm) from three randomly chosen islands per archipelago (a small, a medium and a large one). This resulted in a total of 12 islands sampled and 36 soil cores. For each island, soil cores were placed under insect emergence traps and the number of adult crane flies emerging counted, and counts scaled up to provide an estimate of total abundance per island.

### Link identification

*Field observations of interactions*. Direct observation of interactions in the field was used for plant–pollinator interactions and for these we recorded the flower visitor, the visited plant species and the frequency of each interaction. We were able to directly observe some other interactions in the field, these consisting of predator–prey interactions between the predatory beetle (*C. lateralis*) and crane fly larvae (*S. stictica*), crab (*C. maenas*) and Collembola (*A. maritima*), Collembola (*A. maritima*) and dead amphipod (*G. salinus*), *Littorina* sp and algae, and *H. ulvae* and diatoms (Supplementary Table 1). If an interaction was observed on one island, it was assumed to occur on all islands where the two species co-occurred. Given that many potential interactions were not observed directly, direct observation was complemented by the following three additional methods.

*Gut content analysis*. Fourteen crabs were collected from islands in the four archipelagos and their stomach contents analysed under the microscope. This identified green algae, *Spartina alterniflora*, gastropods and amphipods as prey, the latter from shell and exoskeleton fragments. From all four archipelagos, 20 amphipods were randomly collected. The analysis of their guts under the microscope identified marine macrophytes as the main dietary item.

*Stable isotope analysis*. Plant and animal tissues contain chemical isotopes and the information provided by the isotopic signature—the distribution and relative proportion of certain stable isotopes in the organism's tissue—can be used to draw inferences about an animal's diet and trophic level^8^. Stable isotopes provide insights into trophic relationships between organisms and therefore can be used to develop models of trophic structure. Stable isotopes, especially nitrogen (N) and carbon (C), have become a common and complementary alternative for the study of species' trophic niches. Past and recent studies show that the ratio of ^15^N to ^14^N (expressed as *δ*^15^N) exhibits stepwise enrichment with trophic transfers (average of 3.2‰ for a wide range of species) and thus can be used to estimate an organism's trophic position^8^. Ratios of carbon isotopes (*δ*^13^C), on the other hand, vary substantially among primary producers with different photosynthetic pathways (for example, C3 versus C4 plants), but change little with trophic transfers (0.5–1‰), and thus can be used to infer sources of dietary carbon (for example, is a certain herbivore eating mainly C3 or C4 plants?). The most common plant species on the islands, *S. alterniflora*, is the only C4 plant in our field system and thus its contribution to animal's diet can be inferred easily. It is usual to present *δ*^13^C*–δ*^15^N bi-plots with species (or individuals and populations) plotted based on their mean stable isotope signatures. Relative position of species in this bi-plot space is used to determine aspects of food-web structure such as trophic position and sources of ultimate dietary carbon.

Three species were selected for stable isotope analysis—*G. salinus*, *C. maenas* and *A. maritima*—as they have rather generalist diets and this method enabled us to identify their actual versus their potential diet. Samples were frozen and dried for the analysis and the analysis carried out at the Stable Isotope facility at Rothamstead Research at North Wyke (Okehampton, Devon EX20 2SB, UK). The stable isotope signatures are shown in Supplementary Fig. 1. We compared these results with published literature on stable isotope signatures of primary producers, molluscs, crustaceans and insects that inhabit our field system^9^,^10^,^11^,^12^,^13^,^14^,^15^,^16^,^17^,^18^,^19^,^20^,^21^,^22^,^23^,^24^,^25^,^26^,^27^,^28^,^29^,^30^,^31^. This information altogether, combined with direct observations, gut content analysis (see above) and published studies, showed that: (i) *G. salinus* feeds mainly on marine macrophytes, (ii) *C. maenas* feeds on a variety of sources that range from primary producers to marine invertebrates and (iii) *A. maritima* occupies a higher trophic position and its feeding habits include marine and terrestrial invertebrates, as well as marine macrophytes.

*Evidence from the literature*. We conducted a literature review of studies on the diets of species present in our salt marsh islands^32^,^33^,^34^,^35^,^36^,^37^,^38^,^39^,^40^,^41^,^42^,^43^,^44^,^45^,^46^,^47^,^48^,^49^,^50^,^51^. When possible, we selected studies from the same geographic location. We used published data to determine most of the predator–prey interactions. With a few exceptions (see Supplementary Table 1) predator–prey interactions were difficult to observe directly in the field. Spatial co-occurrence of predator–prey pairs has been found to be a good proxy of predation though^51^. In other words, there is a clear relationship between co-occurrence and feeding behaviour between predators and prey. Here, the spatial co-occurrence of predator–prey pairs identified from the literature was used as a proxy of predation links on each island.

### Calculating the interaction metrics

To examine the structure of the networks we calculated the following metrics: species richness (*S*), number of links (*L*), linkage density or average degree (*L/S*) and connectance (*L/S*^*2*^)^37^. We also measured nestedness (used in the TC analysis, see below), modularity and robustness. Nestedness measures the degree to which the diets of consumers are proper subsets of other, more generalist consumers and it was calculated using the NODF algorithm^48^. Modularity, or compartmentalization, describes the degree to which interactions occur more frequently within modules than between modules. Modularity was estimated using the qualitative algorithm proposed by Newman and Girvan^49^,^50^, which has been already used to identify compartments in food webs^51^,^52^.

The modularity (*Q*) was calculated as^49^,^50^:


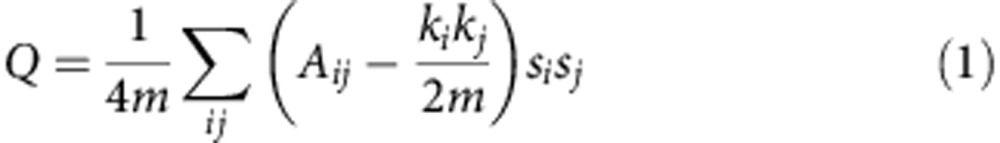


where *m*=½Σ_*i*_ *k*_*i*_ is the total number of edges (that is, links) in the network, *k*_*i*_ and *k*_*j*_ are the degrees of the vertices (that is, species) and *A*_*ij*_ is the number of links between species. In a network with *n* species, for a particular division of the network into two groups *s*_*i*_=1 if species *i* belongs to group 1 and *s*_*i*_=−1 if it belongs to group 2. Each network metric was calculated for each of the 115 island networks.

Robustness measures the topological or structural stability of the food web by simulating how random removal of prey (or predators) induces secondary extinction among the predators (or prey)^53^. Robustness is measured as the area under the curve of the number of species being removed against the number of secondary extinctions and ranges from 0 to 1, with high values representing more robust communities (the number of secondary extinctions is lower). To investigate the robustness of functional group diversity in our salt marsh system, we constructed species–island networks for each archipelago. Robustness was analysed as the changes in these spatial networks to random island removal. We found differences in robustness per site and per functional group (Supplementary Fig. 3). Robustness of functional group diversity was greater for the regional system when compared with single archipelago spatial networks, suggesting that area size is a key factor for the stability of functional group diversity (Supplementary Fig. 3). Robustness was generally higher for primary productivity, habitat provision and herbivory, whereas robustness in pollination was generally low, an effect also reported in agro-ecosystems^15^.

### Contribution of islands to multiple functional groups

Ecosystem functions are usually the product of an interaction between two species, for example, a flowering plant and a flower visitor or a predator and a prey species. These interactions, and the species involved in them, constitute functional groups that are responsible for the provision of functions (for example, plant–pollinator interactions provide the ecosystem function of pollination). We focused on functions that have direct, immediate links to species and their interactions, and determined the number of the corresponding functional groups providing those functions that inhabit each island (Supplementary Table 4). We first calculated the accumulated number of functional groups within and across archipelagos. For example, in the case of two islands, one hosting primary productivity, plant pollination and plant herbivory, and the other island hosting primary productivity and plant herbivory, these two islands would add up to five functional groups together. Second, we estimated the accumulated number of species *S* in each functional group *j* and island *k* (Σ_jk_[*S*_*j*_*, FG*_*k*_]). The contribution of each island *i* to functional group diversity (*FGD*_*i*_) was then calculated as the proportion of species (and their abundances) involved in the functional groups present on island *i* relative to the accumulated number of species (and their abundances) within and across archipelagos (Σ*FGD*_*i*_):


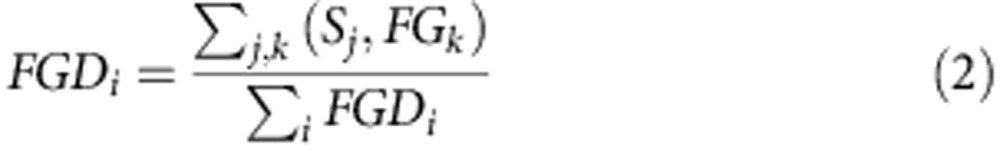


For the estimation of *FGD*_*i*_ using species abundances, *S*_*j*_ is substituted by its corresponding abundance data (all individuals were assumed to contribute equally to ecosystem function). This method allowed us to estimate the mean and s.d. of the number of functional groups hosted per island and the relative contribution of islands to functional group diversity.

### Food-web properties and biogeographic variables

The Trophic Island Biogeography theory^34^ extends classic island biogeography^54^ by combining biogeographic factors related to isolation (that is, distance to mainland) and size (that is, island area) with food-web properties. The theory predicts higher number of links and lower connectance in bigger islands, a prediction successfully tested with a lake and an island food-web data sets^34^. The empirical data sets used by Gravel *et al*.^34^ ranged from 0.8 to 3488.19 ha for the lake data set and from 11 to 25 m in diameter (≈34.5 to≈78.5 m^2^ assuming they are perfectly circular) and 2 to 533 m from the nearest colonization source for the island data set. The island data set in Gravel *et al*.^34^ falls within our island salt marsh system range values, although our area range and distance to mainland are larger and smaller, respectively. The Gravel *et al*.^34^ island data set has a very small sample size (*N*=6 islands) when compared with the 115 salt marsh islands used here. Although there were some statistically significant correlations in our data (Supplementary Table 1), the reported significant relationships between biogeographic variables and food-web properties were not consistent across archipelagos or species origin (terrestrial versus marine).

### The role of individual species in modular networks

In addition to the general modularity analysis, each species within the food webs was assigned a role according to its topological properties^31^,^32^. The role of a species is defined by its position compared with other species in its own module and how it connects to species in other modules. Therefore, the role of a species *i* can be characterized by its standardized within-module degree and its among-module connectivity^31^. We used the method by Oleson *et al*.^32^ to classify each species into peripherals, connectors, module hubs and network hubs. This allows mapping functional groups as a function of species' network role, providing insight into which species topologies are related to which functional groups.

### TC analysis

Following the concept of niche partitioning or resource-use overlap, Poisot *et al*.^35^ recently defined TC as ‘the ‘originality' of a species in a food web relative to the other ones, based on the identity of the species it interacts with'. Using a mathematical model, these authors showed that TC is a general mechanism driving functioning of multi-trophic communities^35^,^36^. TC is based on the nestedness metric and therefore can only be calculated for bipartite consumer–resource interactions. This excludes primary productivity and habitat provision from TC analysis. Plant-pollination networks in our salt marshes comprised only one flowering plant, complicating the calculation of TC, as nestedness cannot be computed reliably in bipartite networks with ≤2 species per trophic level. We performed TC analysis for decomposition, plant-herbivore and prey-predation functional groups, which yielded a total of 131 empirical functional group networks, and calculated TC for additional 54 plant-pollination networks reported in the literature^55^. TC ranges from 0 (no complementarity, that is, food web is entirely nested) to 1 (maximal complementarity, that is, food web is made entirely of unconnected linear chains).

### Statistical analysis

Generalized estimating equations (GEEs) are an extension of generalized linear models when the response variables have been measured repeatedly through time or space, and provide a method that accounts for spatial correlation^30^. Using a parameterized correlation matrix, GEEs take correlations within clusters of sampling units into account, whereas the correlations between clusters are assumed to be zero. In a spatial context, those clusters correspond to geographic regions as long as they are sufficiently distant^56^. The four archipelagos in our salt marsh island system represent these clusters and we assumed that all islands within archipelagos were equally correlated. The violation of this assumption does not pose any significant problem, as the estimates of regression parameters in GEEs are fairly robust against misspecification of the correlation matrix^30^,^57^. The GEE method uses the Quasi-Akaike's Information Criterion for model selection, which is the GEE equivalent to the Akaike's Information Criterion^58^. We used model averaging to obtain the weighted average of the estimates of parameters for models with Quasi-Akaike's Information Criterion ≤2.

All our analyses were carried out using R software^59^.

## 

## Additional information

**How to cite this article:** Montoya, D. *et al*. Functional group diversity increases with modularity in complex food webs. *Nat. Commun.* 6:7379 doi: 10.1038/ncomms8379 (2015).

## Supplementary Material

Supplementary Information Supplementary Figures 1-3, Supplementary Tables 1-4 and Supplementary References.

## Figures and Tables

**Figure 1:**
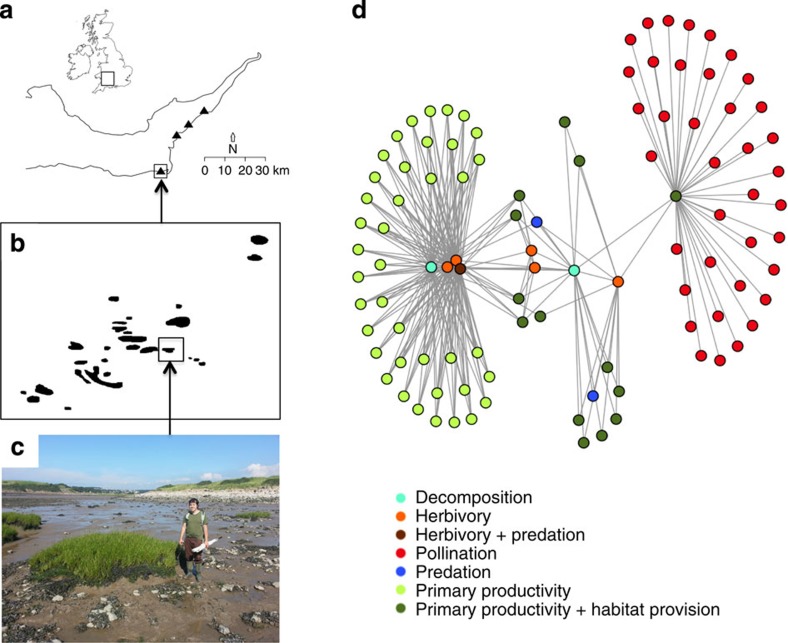
Island salt marsh food webs and functional groups. The distribution of the field sites along the Bristol Channel is shown in **a**. The study system consisted of four archipelagos, one of which is shown in **b**, each comprising 27–31 islands (total=115 islands) (**c**). Each island was sampled individually to generate a total of 115 island food webs. The network of species interactions along with the functional groups is shown in **d**.

**Table 1:** Results of spatial generalized estimating equations.

	Estimate	s.e.	*z*-value	*P*-value
*Regional (*N*=115)*				
Modularity	0.9296	0.0942	9.868	<2 × 10^−16^
Linkage density	0.2246	0.0498	4.513	6.4 × 10^−6^
Distance to mainland	−0.00034	0.00012	2.805	0.005
Links	2.5 × 10^−6^	1.2 × 10^−6^	2.060	0.039
				
*Sand Bay (*N*=31)*				
Modularity	1.369	0.3286	4.168	3.08 × 10^−5^
Linkage density	0.2941	0.1355	2.170	0.030
Distance to mainland	0.00242	0.0010	2.420	0.015
				
*Portishead (*N*=27)*				
Modularity	0.5494	0.2215	2.480	0.013
				
*Poet's Corner (*N*=28)*				
Modularity	1.020	0.2448	4.167	3.08 × 10^−5^
Connectance	9.396	3.778	2.487	0.013
Number of links	2.14 × 10^−6^	1.09 × 10^−6^	2.500	0.020
				
*Steart (*N*=29)*				
Modularity	1.118	0.4479	2.495	0.013
Linkage density	0.1019	0.0482	2.115	0.034
