# Supplementary material for: Functional group diversity increases with modularity in complex food webs
Source: Nat Commun. 2015 Jun 10;6:7379. doi: 10.1038/ncomms8379 (PMC4490355; doi:10.1038/ncomms8379)
Supplement: Supplementary Information — Supplementary Figures 1-3, Supplementary Tables 1-4 and Supplementary References. [file ncomms8379-s1.pdf]

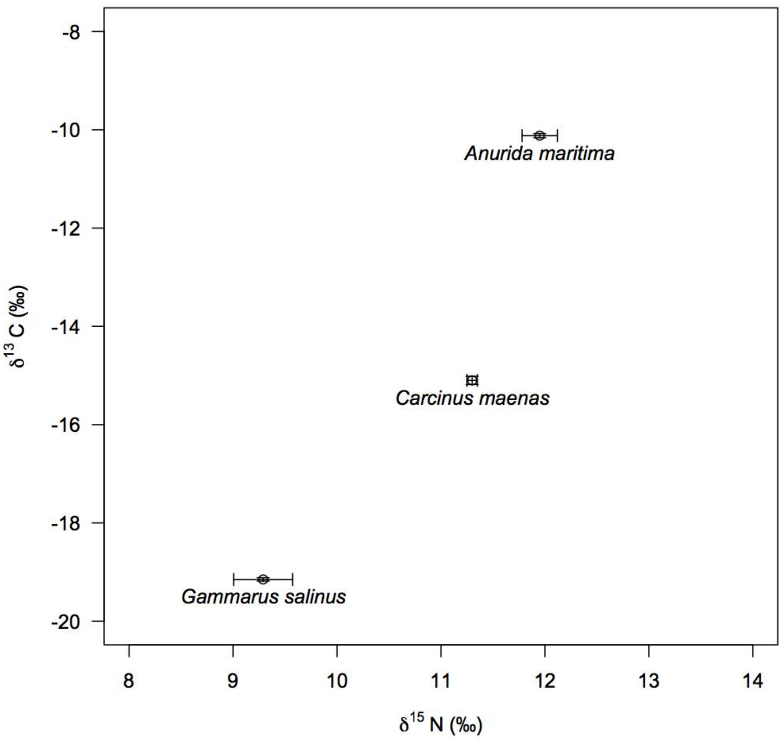

Supplementary Figure 1 | Stable isotope analysis. δ^{13}C versus δ^{15}N data for *Gammarus salinus, Carcinus maenas* and *Anurida maritima*. Error bars (s.d.) are provided.

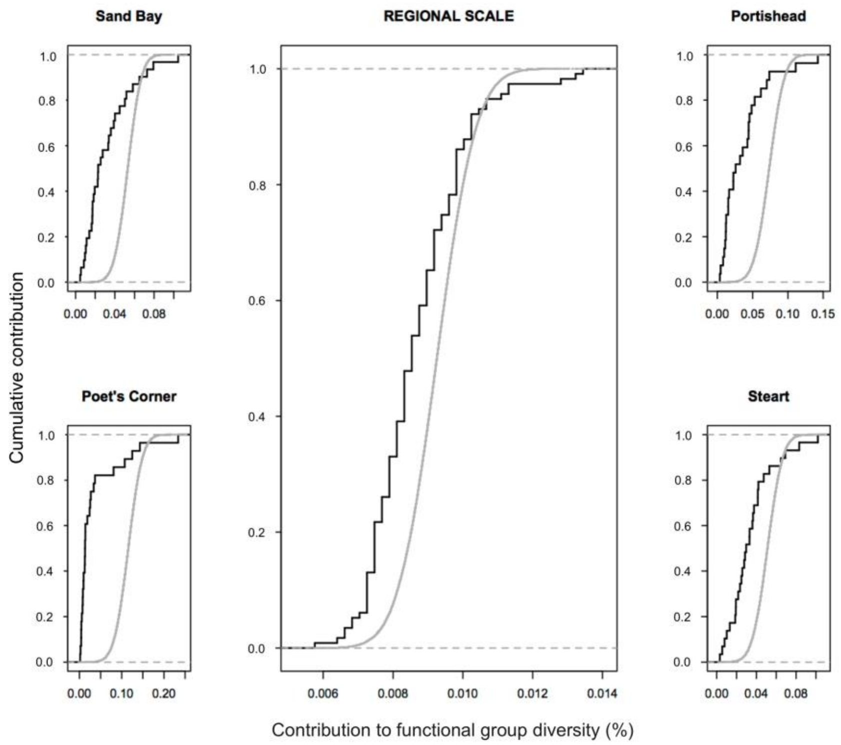

Supplementary Figure 2 | Empirical cumulative distributions of the contribution of each island to functional group diversity. The contribution to functional group diversity, i.e. number of functional groups, is calculated using species richness data. Data is provided for the regional system and each archipelago individually; the black line represents the field data and the grey line represents the empirical cumulative distribution of a normal distribution with the same mean and standard deviation.

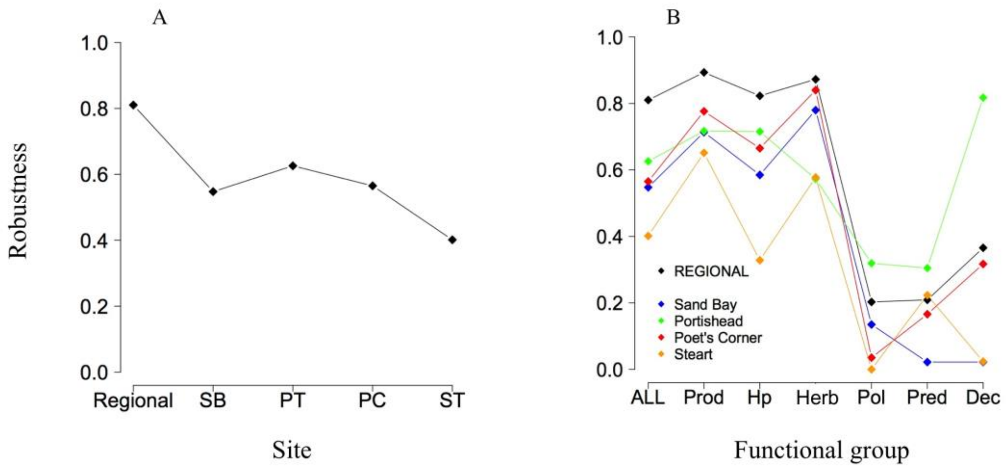

Supplementary Figure 3 | Robustness of functional groups to island removal. (A) Functional robustness per archipelago (Regional = Regional scale; SB = Sand Bay; PT = Portishead; PC = Poet's Corner; ST = Steart). The existence of multiple functional groups is more robust at the regional scale. (B) Robustness per functional group (ALL = All functional groups together; Prod = Primary productivity; Hp = Habitat provision; Herb = Herbivory; Pol = Pollination; Pred = Predation; Dec = Decomposition). Robustness of multiple functional groups varied across archipelagos and functional groups, with pollination and predation being the least robust functional groups.

Species A (Resource species)	Species B (Consumer species)
Aster tripolium	*Apis mellifera*
Aster tripolium	*Bellardia vulgaris*
Aster tripolium	*Bombus lapidarius*
Aster tripolium	*Bombus lucorum*
Aster tripolium	*Bombus pascuorum*
Aster tripolium	*Bombus terrestris*
Aster tripolium	*Ceratinostoma ostiorum*
Aster tripolium	*Dasysyrphus albostriatus*
Aster tripolium	*Episyrphus balteatus*
Aster tripolium	*Eristalinus aeneus*
Aster tripolium	*Eristalis arbustorum*
Aster tripolium	*Eristalis nemorum*
Aster tripolium	*Eristalis tenax*
Aster tripolium	*Eupeodes corollae*
Aster tripolium	*Eupeodes latifasciatus*
Aster tripolium	*Eupeodes luniger*
Aster tripolium	*Fucellia maritima*
Aster tripolium	*Helophilus pendulus*
Aster tripolium	*Lasioglossum calceatum*
Aster tripolium	*Lucilia sericata*
Aster tripolium	*Myrathropa florea*
Aster tripolium	*Neomyia cornicina*
Aster tripolium	*Neomyia viridescens*
Aster tripolium	*Pieris rapae*
Aster tripolium	*Platycherus albimanus*
Aster tripolium	*Platycherus manicatus*
Aster tripolium	*Platycherus scutatus*
Aster tripolium	*Pogonus chalceus*
Aster tripolium	*Sarcophaga canaria*
Aster tripolium	*Sarcophaga subvicina*
Aster tripolium	*Scatophaga litorea*
Aster tripolium	*Scatophaga stercoraria*
Aster tripolium	*Siphona geniculata*
Aster tripolium	*Syritta pipiens*
Aster tripolium	*Syrphus ribesii*
Aster tripolium	*Syrphus torvus*
Aster tripolium	*Syrphus vitripensis*
Aster tripolium	*Vespula vulgaris*
Symplecta stictica	*Cillenus lateralis*
Carcinus maenas	*Anurida maritima*
Gammarus salinus	*Anurida maritima*
Ulva intestinalis	*Littorina littorea*
Ulva lactuca	*Littorina littorea*
Fucus vesiculosus	*Littorina littorea*
Vaucheria spp.	*Littorina littorea*
Ulva intestinalis	*Littorina obstusata*
Ulva lactuca	*Littorina obstusata*
Fucus vesiculosus	*Littorina obstusata*
Vaucheria spp.	*Littorina obstusata*

Supplementary Table 1 | List of interactions directly observed in the field

	Common name	Primary productivity	Habitat provision	Herbivory	Pollination	Predation	Decomposition	**Functions / species**
Achnanthes brevipes	Diatom	✓						1
Achnanthes sp.	Diatom	✓						1
Actinoptychus sp.	Diatom	✓						1
Amphiprora alata	Diatom	✓						1
Amphiprora surirelloides	Diatom	✓						1
Amphora sp.	Diatom	✓						1
Anurida maritima	Collembola						✓	1
Apis mellifera	Insect pollinator				✓			1
Aster tripolium	Terrestrial plant	✓	✓					2
Bacillaria sp.	Diatom	✓						1
Bellardia vulgaris	Insect pollinator				✓			1
Bombus lapidarius	Insect pollinator				✓			1
Bombus lucorum	Insect pollinator				✓			1
Bombus pascuorum	Insect pollinator				✓			1
Bombus terrestris	Insect pollinator				✓			1
Carcinus maenas	Decapod			✓		✓		2
Ceratinostoma ostiorum	Insect pollinator				✓			1
Cillenus lateralis	Predator beetle					✓		1
Cocconeis cf. disculus	Diatom	✓						1
Cocconeis sp. 1	Diatom	✓						1
Cocconeis sp. 2	Diatom	✓						1
Cochlearia anglica	Terrestrial plant	✓	✓					2
Coscinodiscus sp. 1	Diatom	✓						1
Coscinodiscus sp. 2	Diatom	✓						1
Cyclotella sp.	Diatom	✓						1
Cylindrotheca sp.	Diatom	✓						1
Dasysyrphus albostriatus	Insect pollinator				✓			1
Diploneis sp.	Diatom	✓						1
Episyrphus balteatus	Insect pollinator				✓			1
Eristalinus aeneus	Insect pollinator				✓			1
Eristalis arbustorum	Insect pollinator				✓			1
Eristalis nemorum	Insect pollinator				✓			1
Eristalis tenax	Insect pollinator				✓			1
Eupeodes corollae	Insect pollinator				✓			1
Eupeodes latifasciatus	Insect pollinator				✓			1
Eupeodes luniger	Insect pollinator				✓			1
Fucellia maritima	Insect pollinator				✓			1
Fucus vesiculosus	Brown algae	✓	✓					2
Gammarus salinus	Amphipod			✓				1
Glaux maririma	Terrestrial plant	✓	✓					2
Gomphonema sp.	Diatom	✓						1
Gyrosigma sp.	Diatom	✓						1
Helophilus pendulus	Insect pollinator				✓			1
Hydrobia ulvae	Gastropod snail			✓				1
Lasioglossum calceatum	Insect pollinator				✓			1
Lekanosphaera rugicauda	Isopod						✓	1
Linyphiidae sp.	Predator spider					✓		1
Littorina littorea	Gastropod snail			✓				1
Littorina obtusata	Gastropod snail			✓				1
Lucilia sericata	Insect pollinator				✓			1
Myrathropa florea	Insect pollinator				✓			1

	Common name	Primary productivity	Habitat provision	Herbivory	Pollination	Predation	Decomposition	**Functions / species**
Navicula gregaria	Diatom	✓						1
Navicula pygmaea	Diatom	✓						1
Navicula sp. 1	Diatom	✓						1
Navicula sp. 2	Diatom	✓						1
Navicula sp. 3	Diatom	✓						1
Navicula sp. 4	Diatom	✓						1
Navicula sp. 5	Diatom	✓						1
Neomyia cornicina	Insect pollinator				✓			1
Neomyia viridescens	Insect pollinator				✓			1
Nitzschia epithemioides	Diatom	✓						1
Nitzschia hungarica	Diatom	✓						1
Nitzschia sigma	Diatom	✓						1
Nitzschia sp. 1	Diatom	✓						1
Nitzschia sp. 2	Diatom	✓						1
Opephora sp.	Diatom	✓						1
Pieris rapae	Insect pollinator				✓			1
Pinnularia sp.	Diatom	✓						1
Plagiogramma sp.	Diatom	✓						1
Plantago maritima	Terrestrial plant	✓	✓					2
Platycheirus manicatus	Insect pollinator				✓			1
Platycherus albimanus	Insect pollinator				✓			1
Platycherus scutatus	Insect pollinator				✓			1
Pleurosigma sp.	Diatom	✓						1
Pogonus chalceus	Insect pollinator				✓			1
Pucinella maritima	Terrestrial plant	✓	✓					2
Raphoneis sp.	Diatom	✓						1
Salicornia europaea	Terrestrial plant	✓	✓					2
Sarcophaga canaria	Insect pollinator				✓			1
Sarcophaga subvicina	Insect pollinator				✓			1
Scatophaga litorea	Insect pollinator				✓			1
Scatophaga stercoraria	Insect pollinator				✓			1
Scoliopleura sp.	Diatom	✓						1
Siphona geniculata	Insect pollinator				✓			1
Spartina anglica	Terrestrial plant	✓	✓					2
Spergularia marina	Terrestrial plant	✓	✓					2
Suaeda maritima	Terrestrial plant	✓	✓					2
Surirella sp. 1	Diatom	✓						1
Surirella sp. 2	Diatom	✓						1
Symplecta stictica	Crane fly			✓				1
Syritta pipiens	Insect pollinator				✓			1
Syrphus ribesii	Insect pollinator				✓			1
Syrphus torvus	Insect pollinator				✓			1
Syrphus vitripensis	Insect pollinator				✓			1
Triglochin maritima	Terrestrial plant	✓	✓					2
Tryblionella sp.	Diatom	✓						1
Ulva intestinalis	Green algae	✓	✓					2
Ulva lactuca	Green algae	✓	✓					2
Unidentified Sp 1	Diatom	✓						1
Unidentified Sp 2	Diatom	✓						1
Vaucheria spp.	Yellow-green algae	✓	✓					2
Vespula vulgaris	Insect pollinator				✓			1
Species / function		54	14	6	38	3	2	117

Supplementary Table 2 | List of species and functional groups

			Species richness	Links	Connectance	Average degree	Maximum Degree	Modularity
**REGIONAL**								
	**All species**	*vs* ↑Size	↑	↑	-	-	-	-
		vs ↑Distance	↓	-	-	-	↑	↓
	**Marine**	*vs* ↑Size	-	-	-	-	-	-
		vs ↑Distance	↑	-	-	-	-	-
	**Terrestrial**	*vs* ↑Size	↑	↑	-	-	-	-
		vs ↑Distance	↓	↓	↑	↓	↓	↓
**SAND BAY**								
	**All species**	*vs* ↑ Size	-	↑	-	-	-	-
		vs ↑Distance	-	-	-	-	-	-
	**Marine**	*vs* ↑ Size	-	-	-	-	-	-
		vs ↑Distance	-	-	-	-	-	-
	**Terrestrial**	*vs* ↑ Size	-	-	-	-	-	-
		vs ↑Distance	-	-	-	-	-	-
**PORTISHEAD**								
	**All species**	*vs* ↑ Size	↑	↑	-	-	-	-
		vs ↑Distance	↓	-	-	-	-	↓
	**Marine**	*vs* ↑ Size	-	-	-	-	-	-
		vs ↑Distance	-	-	-	-	-	-
	**Terrestrial**	*vs* ↑ Size	↑	↑	↓	-	↑	↑
		vs ↑Distance	↓	↓	↑	-	↓	↓
**POET'S CORNER**								
	**All species**	*vs* ↑ Size	↑	↑	-	-	-	↑
		vs ↑Distance	-	-	↑	-	-	↓
	**Marine**	*vs* ↑ Size	-	-	-	-	-	-
		vs ↑Distance	-	↑	-	↑	-	-
	**Terrestrial**	*vs* ↑ Size	↑	↑	↓	↑	↑	↑
		vs ↑Distance	↓	↓	↑	↓	↓	↓
**STEART**								
	**All species**	*vs* ↑ Size	-	↑	-	↑	-	-
		vs ↑Distance	-	-	-	-	-	-
	**Marine**	*vs* ↑ Size	-	↑	-	↑	-	-
		vs ↑Distance	-	-	-	-	-	-
	**Terrestrial**	*vs* ↑ Size	-	-	-	-	-	-
		vs ↑Distance	-	-	-	-	-	-

Supplementary Table 3 | Summary results for linear models between island food web properties and biogeographic variables. Arrow orientation indicates whether significant correlations ($p<0.05$) are positive (up) or negative (down). The absence of arrows indicates not significant relationships.

	Island size	Distance to mainland	Number of species	Number of functions	Abundance	Links	Connectance	Average degree	Modularity
Island size	1	0.0008	0.0366	0.0300	0.8169	0.0020	3.94E-05	0.0007	0.0185
Distance to mainland	0.0008	1	0.0954	0.2137	3.55E-05	0.0069	0.0230	0.0004	0.1816
Number of species	0.0366	0.0954	1	0.3901	0.0484	0.4175	0.0041	0.1672	0.1587
Number of functions	0.0300	0.2137	0.3901	1	0.0467	0.3262	0.0661	0.2119	0.2141
Abundance	0.8169	3.55E-05	0.0484	0.0467	1	0.0037	0.0001	0.0014	0.0267
Links	0.0020	0.0069	0.4175	0.3262	0.0037	1	0.6067	0.9090	0.0214
Connectance	3.94E-05	0.0230	0.0041	0.0661	0.0001	0.6067	1	0.8636	0.2440
Average degree	0.0007	0.0004	0.1672	0.2119	0.0014	0.9090	0.8636	1	0.0951
Modularity	0.0185	0.1816	0.1587	0.2141	0.0267	0.0214	0.2440	0.0951	1

Supplementary Table 4 | Correlation matrix between island food web properties and biogeographic variables (N = 115)

Supplementary references

1. Bassindale, R. Studies on the biology of the Bristol Channel: XI. The physical environment and intertidal fauna of the southern shores of the Bristol Channel and Severn Estuary. *J. Ecol.* **31**, 1-29 (1943).
2. Bassindale, R. The distribution of amphipods in the Severn Estuary and Bristol Channel. *J. Anim. Ecol*. **11**, 131-144 (1942).
3. Collins, N. R. & Williams, R. Zooplankton of the Bristol Channel and Severn Estuary. The distribution of four copepods in relation to salinity. *Mar. Biol.* **64**, 273-283 (1981).
4. Hamilton, P. The circulation of the Bristol Channel. *Geophys. J. R. Astr. Soc*. **32**, 409-422 (1973).
5. Stephens, C. V. A three-dimensional model for tides and salinity in the Bristol Channel. *Cont. Shelf Res.* **6**, 531-560 (1986).
6. Uncles, R. J. Hydrodynamics of the Bristol Channel. *Mar. Pollut. Bull.* **15**, 47-53 (1983).
7. Uncles, R. J. Physical properties and processes in the Bristol Channel and Severn Estuary. *Mar. Pollut. Bull.* **61**, 5-20 (2010).
8. Michener, R. & Lajtha, K. (Eds). *Stable Isotopes in Ecology and Environmental Science* (Willey-Blackwell, Oxford, 2007).
9. Søreide, J. E. & Nygård, H. Challenges using stable isotopes for estimating trophic levels in marine amphipods. *Polar. Biol*. **35**, 447-453 (2012).
10. Nyssen, F. et al. A stable isotope approach to the eastern Weddell Sea trophic web: focus on benthic amphipods. *Polar Biol*. **25**, 280-287 (2002).
11. Farlin, J.P. et al. Functional diversity in amphipods revealed by stable isotopes in an eelgrass ecosystem. *Mar. Ecol.-Prog. Ser*. **420**, 277–281 (2010).
12. Limen, H., van Overdijk, C. D. A. & MacIsaac, H. J. Food partitioning between the amphipods *Echinogammarus ischnus*, *Gammarus fasciatus*, and *Hyalella azteca* as revealed by stable isotopes. *J. Great Lakes Res*. **31**, 97-104 (2005).
13. Hart, E. A. & Lovvorn, J. R. Interpreting stable isotopes from macroinvertebrate food webs in saline wetlands. *Limnol. Oceanogr*. **47**, 580–584 (2002).
14. Unkovich, M. et al (Eds). *Stable Isotope Techniques in the Study of Biological Processes and Functioning of Ecosystems* (Kluwer Academic Publishers, Dordrecht, 2001).

15. Adin, R. & Riera, P. Preferential food source utilization among stranded macroalgae by *Talitrus saltator* (Amphipod, Talitridae): a stable isotopes study in the northern coast of Brittany (France). *Estuar. Coast. Shelf S.* **56**, 91-98 (2003).

16. Attrill M.J. et al. Oligochaetes as a possible entry route for terrigenous organic carbon into estuarine benthic food webs. *Mar. Ecol. Prog. Ser*. **384**, 147−157 (2009).

17. Riera P. et al. Determination of food sources for benthic invertebrates in a salt marsh (Aiguillon Bay, France) by carbon and nitrogen stable isotopes: importance of locally produced sources. *Mar. Ecol. Prog. Ser*. **187**, 301–307 (1999).

18. Watts, A. J. R. et al. Does seabird carrion contribute to the diet of the shore crab *Carcinus maenas* on the isle of May, Scotland? An isotopic perspective. *J. Mar. Biol. Assoc. UK* **91**, 1459-1464 (2011).

19. Sarà, G. et al. Sources of organic matter for intertidal consumers on *Ascophyllum*-shores (SW Iceland): a multi-stable isotope approach. *Helgoland. Mar. Res*. **61**, 297-302 (2007).

20. Vafeiadou, A. M. et al. Food sources of macrobenthos in an estuarine seagrass habitat (*Zostera noltii*) as revealed by dual stable isotope signatures. *Mar. Biol*. **160**, 2517–2523 (2013).

21. Haines, E. B. Stable carbon isotope ratios in the biota, soils and tidal water of a Georgia salt marsh. *Estuar. Coast. Shelf S*. **4**, 609-616 (1976).

22. Haines, E. B. & Montague, C. L. Food sources of estuarine invertebrates analysed using $^{13}C/^{12}C$ ratios. *Ecology* **60**, 48-56 (1979).

23. Peterson, B. J. & Howarth, R. W. Sulfur, carbon, and nitrogen isotopes used to trace organic matter flow in the salt-marsh estuaries of Sapelo Island, Georgia. *Limnol. Oceanogr*. **32**, 1195-1213 (1987).

24. Peterson, B. J. & Fry, B. Stable isotopes in ecosystem studies. *Ann. Rev. Ecol. Syst*. **18**, 293-320 (1987).

25. Cloern, J. E., Canuel, E. A. & Harris, D. Stable carbon and nitrogen isotope composition of aquatic and terrestrial plants of the San Francisco Bay estuarine system. *Limnol. Oceanogr*. **47**, 713-729 (2002).

26. Kwak, T. J. & Zedler, J. B. Food web analysis of southern California coastal wetlands using multiple stable isotopes. *Oecologia* **110**, 262-277 (1997).

27. Bergamino, L., Lercari, D. & Defeo, O. Terrestrial trophic subsidy in sandy beaches: evidence from stable isotope analysis in organic matter sources and isopod *Excirolana armata*. *Aquat. Ecol*. **14**, 129-134 (2012).
28. Henninger, T. O. Aspects of the ecology and biology of the isopod, Exosphaerona hylocoetes (Barnard, 1940) in three temporarily open/closed southern African estuaries. PhD thesis (2008).
29. Rossi, L., Costantini, M. L. & Brilli, M. Does stable isotope analysis separate transgenic and traditional corn (*Zea mays* L.) detritus and their consumers? *Appl. Soil Ecol*. **35**, 449-453 (2007).
30. Platner, C. et al. Trophic diversity in a Mediterranean food web – Stable isotope analysis of an ant community of an organic citrus grove. *Basic Appl. Ecol*. **13**, 587-596 (2012).
31. Sturaro, N. et al. Trophic diversity of idoteids (Crustacea, Isopoda) inhabiting the *Posidina oceanica* litter. *Mar. Biol*. **157**, 237-247 (2010).
32. Andersson, S., Persson, M., Moksnes, P.-O. & Baden, S. The role of the amphipod *Gammarus locusta* as a grazer on macroalgae in Swedish seagrass meadows. *Mar. Biol*. **156**, 969-981 (2009).
33. Arrontes, J. Diet, food preference and digestive efficiency in intertidal isopods inhabiting macroalgae. *J. Exp. Mar. Biol. Ecol*. **139**, 231-249 (1990).
34. Baeta, A., Cabral, H. N., Marques, J. C. & Pardal, M. A. Feeding ecology of the green crab *Carcinus maenas* (L. 1758) in a temperate estuary, Portugal. *Crustaceana* **79**, 1181-1193 (2006).
35. Baird, D., Asmus, H. & Asmus, R. Energy flow of a boreal intertidal ecosystem, the Sylt-Rømø Bight. *Mar. Ecol. Prg. Ser*. **279**, 45-61 (2004).
36. Baird, D., Asmus, H. & Asmus, R. Trophic dynamics of eight intertidal communities of the Sylt-Rømø Bight ecosystem, northern Wadden Sea. *Mar. Ecol. Prg. Ser*. **351**, 25-41 (2007).
37. Crothers, J. H. The Biology of the Shore Crab. *Fls. Stud*. **3**, 263-274 (1970).
38. De Troch, M., Steinarsdóttir, M. B., Chepurnov, V. & Ólafsson, E. Grazing on diatoms by harpacticoid copepods: species-specific density-dependent uptake and microbial gardening. *Aquat. Microb. Ecol.* **39**, 135-144 (2005).
39. Finch, O.-D., Krummen, H., Plaisier, F. & Schultz, W. Zonation of spiders (Araneae) and carabid beetles (Coleoptera: Carabidae) in island salt marshes at the North Sea coast. *Wetlands Ecol. Manage.* **15**, 207-228 (2007).
40. Fish, J. D. & Fish, S. *A Student's Guide to the Seashore* (Cambridge University Press, Cambridge, 2011).

41. Goecker, M. E. & Kåll, S. E. Grazing preferences of marine isopods and amphipods on three prominent algal species of the Baltic Sea. *J. Sea. Res*. **50**, 309-314 (2003).
42. Johnston, D. & Freeman, J. Dietary preference and digestive enzyme activities as indicators of trophic resource utilization by six species of crab. *Biol. Bull*. **208**, 36-46 (2005).
43. McKinney, R.A., Glatt, S.M. & Williams, S.R. Allometric length-weight relationships for benthic prey of aquatic wildlife in coastal marine habitats. *Wildlife Biol*. **10**, 241-249 (2004).
44. Pihl, L. Food selection and consumption of mobile epibenthic fauna in shallow marine areas. *Mar. Ecol. Prog. Ser*. **22**, 169-179 (1985).
45. Rangeley, R. W. & Thomas, M. L. H. Predatory behaviour of juvenile shore crab *Carcinus maenas* (L.). *J. Exp. Mar. Biol. Ecol*. **108**, 191-197 (1987).
46. Underwood, G. J. C. Microphytobenthos and phytoplankton in the Severn Estuary, UK: Present situation and possible consequences of a tidal energy barrage. *Mar. Pollut. Bull.* **61**, 83-91 (2010).
47. Underwood, G. J. C., Thomas, J. D. & Baker, J. H. An experimental investigation of interactions in snail-macrophyte-epiphyte systems. *Oecologia* **91**, 587-595 (1992).
48. Vince, S. W., Valiela, I. & Teal, J. M. An experimental study of the structure of herbivorous insect communities in a salt marsh. *Ecology* **62**, 1662-1678 (1981).
49. Ropes, J. W. The feeding habits of the green crab, Carcinus maenas (L.). *Fish. B-NOAA* **67**, 183-203 (1968).
50. Sahan, E. S. et al. Community structure and seasonal dynamics of diatom biofilms and associated grazers in intertidal mudflats. *Aquat. Microb. Ecol.* **47**, 253-266 (2007).
51. Bell J. R., King, A., Bohan, D. A. & Symondson, W. O. C. Spatial co-occurrence networks predict the feeding histories of polyphagous predators at field scales. *Ecography* **33**, 64-72 (2010).
